# Modulation of astrocyte activity and improvement of oxidative stress through blockage of NO/NMDAR pathway improve posttraumatic stress disorder (PTSD)‐like behavior induced by social isolation stress

**DOI:** 10.1002/brb3.2620

**Published:** 2022-05-23

**Authors:** Hua Li, Arash Mohammadi Tofigh, Azita Amirfakhraei, Xuan Chen, Michael Tajik, Dongwei Xu, Saeid Motevalli

**Affiliations:** ^1^ Department of Neurology 3201 Hospital Xi'an Jiaotong University Health Science Center Hanzhong China; ^2^ Department of General Surgery School of Medicine Shahid Beheshti University of Medical Sciences Tehran Iran; ^3^ Department of Psychology Bandar Abbas Branch Islamic Azad University Bandar Abbas Iran; ^4^ Department of Neurosurgery The 78th Group Army Hospital of Chinese PLA Mudanjiang China; ^5^ Department of Pharmacology School of Medicine Shahid Beheshti University of Medical Sciences Tehran Iran; ^6^ Faculty of Social Sciences & Liberal Arts Department of Psychology UCSI University Kuala Lumpur Malaysia

**Keywords:** astrocyte, fear memory, oxidative stress, PTSD, social isolation stress

## Abstract

**Background:**

It has been well documented that social isolation stress (SIS) can induce posttraumatic stress disorder (PTSD)‐like behavior in rodents, however, the underlying mechanism is remained misunderstood. In the current study, we aimed to elucidate the role of NO/NMDAR pathway in PTSD‐like behavior through modulating of astrocyte activity and improvement of oxidative stress.

**Methods:**

Male NMRI mice were used to evaluate the memory function by using Morris water maze (MWM) and fear memory extinction by using freezing response. We used MK‐801 (NMDAR‐antagonist), L‐NNA (NOS‐inhibitor), NMDA (NMDAR‐agonist), and L‐arginine (NO‐agent) to find a proper treatment. Also, immunohistochemistry, RT‐PCR, and oxidative stress assays were used to evaluate the levels of astrocytes and oxidative stress. We used five mice in each experimental task.

**Results:**

Our results revealed that SIS could induce learning and memory dysfunction as well as impairment of fear memory extinction in MWM and freezing response tests, respectively. Also, we observed that combined treatment including blockage of NOS (by L‐NNA, 0.5 mg/kg) and NMDAR (by MK‐801, 0.001 mg/kg) at subeffective doses could result in improvement of both memory and fear memory. In addition, we observed that SIS significantly increases the GFAP expression and astrocyte activity, which results in significant imbalance in oxidative stress. Coadministration of MK‐801 and L‐NNA at subeffective doses not only decreases the expression of GFAP, but also regulates the oxidative stress imbalance

**Conclusion:**

Based on these results, it could be hypothesized that blockage of NO/NMDAR pathway might be a novel treatment for PTSD‐like behavior in animals by inhibiting the astrocyte and regulating oxidative stress level.

## Graphical abstract

Schematic of astrocyte activation induced by social isolation stress and increase in oxidative stress as well as modulation of NO/NMDAR pathway in posttraumatic stress disorder (PTSD)‐like behavior.

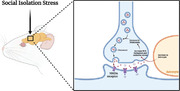

## 1 INTRODUCTION

Posttraumatic stress disorder (PTSD) following acute stress, trauma brain injury (TBI), etc. is a mental illness and causing a serious decline in the quality of life in the general population (Byers et al., 2010; Garcia‐Leal et al., 2010; Lucke‐Wold et al., 2018). Studies have confirmed that experiencing stress in early stages of life can considerably affect neurodevelopment, resulting in a higher risk of impaired fear memory extinction and learning disabilities (Lucke‐Wold, 2011; Lupien et al., 2007, 2009). Previous studies suggested that social isolation stress (SIS) is a valid model of PTSD in mice (Pinna, 2019); therefore, studying SIS animal models could shed light on the pathways behind fear memory extinction and might lead to novel therapeutic approaches.

Although, it is not known precisely that how the fear memory extinction pathway works in the learning and memory process, it is assumed that the hippocampus and amygdala are the main contributor in this system (Sotres‐Bayon et al., 2006). Emerging evidence has demonstrated that hippocampal N‐methyl‐D‐ receptor (NMDAR) and substream signaling pathways may be involved in the pathophysiological mechanism of fear memory and impairment of fear memory extinction (Bevilaqua et al., 2005). On the other hand, it has been suggested that elevated levels of nitric oxide (NO, synthesizes by NOS enzyme) in the regions of the brain related to memory and emotion, such as hippocampus, can cause PTSD‐like behavior in animals (Oosthuizen et al., 2005). NOS activity is found to be involved in regulating behavioral, emotional, learning, and memory processes (Gulpinar & Yegen, 2004). Interestingly, it has been stated that the concentration of NO in the brain is linked to an increase in the activation of the NMDAR (Bergstrom et al., 2015). Based on this research, it has been demonstrated that NOS inhibitors increase the effectiveness of NMDAR inhibitors in reducing glutamate release in the cortex (Bergstrom et al., 2015). Thus, in this study, we used a combination of NOS and NMDAR inhibitors to evaluate a possible effect on fear memory and evaluate the pathophysiology of PTSD‐like behavior in SIS.

Astrocytes are widely present in the central nervous system (CNS), and they play a much more active role in brain physiology under a chronic stress condition (Kol & Goshen, 2021). Although, the role of astrocytes is not well‐documented in response to chronic stress, it has been suggested that they may directly modify synaptic activity by the release of active molecules, called gliotransmitters (Araque et al., 2014). Glial fibrillary acidic protein (GFAP) is a cytoskeletal protein of astrocytes, which typically used as an important marker of astrocyte activation (Yetimler et al., 2020). Many studies have found that astrocytes can be activated following chronic stress (Du Preez et al., 2020, 2021). In this regard, they suggested that overactivation of astrocytes may result in excess oxidative stress, and subsequently increase neuroinflammation and brain damage (Chen et al., 2020). However, the exact underlying mechanism remains misunderstood.

Oxidative stress refers to the imbalance between oxidation and antioxidation, which refers to higher levels of reactive oxygen species (ROS), large amount of protein, lipid, and nucleic acid (DNA) in cellular matrix, due to cell damage (Haj‐Mirzaian et al., 2019; Zhang et al., 2020). Since the brain's tissue contains a large number of unsaturated fatty acids, it is highly sensitive to oxidative damage. In addition, previous studies showed that SIS can significantly increase production of ROS and decrease antioxidant agents such as glutathione (GSH) in the brain (Seydi et al., 2021).

On the other hand, recent studies have shown that astrocyte can play a pivotal role in fear memory and modulating the NMDAR‐dependent long‐term memory (Li et al., 2020; Navarrete et al., 2019). Since induction of fear memory and also impaired extinction of traumatic memory is one of the hallmark symptoms of PTSD, development of new and novel effective treatment in a valid animal model such as SIS can significantly promote our understanding of mechanisms of PTSD. Therefore, in the current study, we aimed to elucidate the role of SIS in NO/NMDAR signaling pathway‐induced fear memory and PTSD‐like behavior through modulating of astrocyte activity and improvement of oxidative stress.

## 2 MATERIALS AND METHODS

### 2.1 Animals and housing conditions

Male NMRI mice weighing 14–16 g on postnatal day (PND) 20–24 were purchased from the Pasteur Institute (Tehran, Iran). Animals were assigned to two opposing conditions including social condition (SC) and isolated condition (IC) for a period of 5 weeks (Hajmirzaeyian et al., 2021; Haj‐Mirzaian et al., 2018). Mice were housed in plexiglas boxes (25 cm × 25 cm × 15 cm) according to the following protocol: four mice were placed in each box for the SC, and mice were located individually in each cage for the IC groups. All animals had free access to food and water under the standard laboratory conditions (temperature: 22 ± 2°C, humidity: 50 ± 10%, 12‐h day/night cycle). The isolated mice had no physical or visual contact with other conspecifics. In order to minimize handling and social interaction, the cages of animals were cleaned weekly by the same experimenter. All assessments were performed during the period between 10:00 am and 03:00 pm after 5 weeks of housing. Before starting the behavioral experiments, each mouse had been handled by the experimenter for the duration of 2 min in two sessions with time intervals of 10 min in order to avoid experimenter‐induced anxiety. All parts of the study were carried out in accordance with National Institutes of Health (NIH) Guide for the Care and Use of laboratory animals (NIH publication No. 86‐23, Eighth Ed.) and institutional and governmental concerns for animal care and use. All efforts were made to minimize the number of animals used and their suffering. Due to differences between male and female mice regarding to menstrual and hormonal changes we used male mice in this study.

### 2.2 Drugs

Following drugs were used: MK‐801 (NMDAR antagonist), aminoguanidine (AG), a selective iNOS (nitric oxide synthesase) inhibitor, Nω‐Nitro‐l‐arginine (L‐NNA), a nonselective NOS inhibitor, 7‐nitro indazole (7‐NI), a selective nNOS inhibitor, and L‐arginine, as a NO agent (All drugs were purchased from Sigma, St Louis, MO, USA). All drugs dissolved in saline except for 7‐NI which dissolved in Tween80 (1%) solution; also, all drugs injected through intraperitoneal (i.p.) route. All drugs (monotherapy or combined therapy) were administrated daily during the 4‐week of social isolation stress.

### 2.3 Experimental design

In the current study, all selected animals were divided in two major groups of IC and SC. Each group was divided into eight subgroups that received one of the following treatment: 1‐MK‐801 0.001 mg/kg, 2‐MK‐801 0.005 mg/kg, 3‐NMDA 15 mg/kg, 4‐NMDA 75 mg/kg, 5‐L‐NNA 0.5 mg/kg, 6‐L‐NNA 1 mg/kg, 7‐L‐arginine 50 mg/kg, and 8‐L‐arginine 100 mg/kg (doses were chosen based on previously published reports; Hajmirzaeyian et al., 2021; Haj‐Mirzaian et al., 2018). All mice in each group were scheduled to receive a daily dose for 28 days continues days (4 weeks of SIS) and all drugs were injected intraperitoneally (i.p).

After a 4‐week period of SIS and treatment learning memory of rodents were assessed using MWM and a fear‐aggravated test namely passive avoidance test, we collected the whole hippocampal tissues for further assessments. After the tests were completed data were analyzed and based on data, we selected MK‐801 at 0.001 mg/kg and L‐NNA at 0.5 mg/kg as subeffective dose. Then the study was continued by another five more socially isolated animals (a combined treatment group). Again, all behavioral tasks were assessed in combined treatment group. Then hippocampal tissues were collected and evaluated by immunohistochemistry and oxidative stress assay. In each experimental task we used five mice.

### 2.4 Morris water maze

After 4 weeks of SIS, the learning and memory ability of mice in each group was tested by Morris water maze (MWM) experiment. Before the start of the experiment, the mice in each group were brought to the water maze laboratory to familiarize with the environment. MWM is a large circular pool with a diameter of 120 cm and a height of 50 cm. Theoretically, the pool was divided into 4 quadrants, and a transparent glass platform with a diameter of 9 cm and the height of 11 cm was placed in the center of one of the quadrants. After the platform is placed, pour water (25 ± 1°C) into the pool (1 cm higher than the platform). Then, pour milk powder into the water and stir evenly so that the mice cannot see the platform (Figure S1a). Also, there is a smart camera (infrared) installed above the pool, and the laboratory's electricity.

Before the water maze experiment, the mice head was marked with red dye to facilitate the imaging during the experiment. MWM is mainly divided into positioning navigation experiment (acquired training), space exploration experiment (exploration training), and visual platform experiment.

#### 2.4.1 Positioning navigation experiment (acquired training)

(1) Put the mice into the water toward the wall of the pool, and randomly select one of the four quadrants to start, and the times required for the mice to find the platform from the moment of entering the water were recorded (escape latency, in seconds). We set the total time to 120 s, and if the mice could not find the platform within 120 s, the escape latency time is recorded as 120 s. At this time, we guided the mice to find the platform and let the mice stayed on the platform for 20 s. (2) Each mice trained four times a day (each time it needed to start from different quadrants), the two training drills separated by 15–20 min, and the entire training period was 5 days. On the second day after the end of the last training period, the platform placed in the pool was removed, and the exploration training with a total of 120 s was started. We put the mice into the water from the quadrant, which was opposite to the quadrant where the original platform was located. Record the percentage of time the mice spent in the target quadrant (the quadrant where the platform was originally placed) and the time that mice were crossed the original platform was used as a test indicator of the mice spatial memory ability.

#### 2.4.2 Visual platform experiment

In order to exclude the influence of the exercise ability of the mice in above experiments, after the exploratory training, a visual platform experiment was done. At this time, the platform was placed in the center of the quadrant opposite to the quadrant where the platform is located during the acquired training, and the platform was 2 cm above the water surface. We put the mice into the water from the contralateral quadrant of the platform, and we recorded the swimming speed of the mice and the time reaching the platform (escape latency).

### 2.5 Freezing response time test

In this study, we used shuttle box only for evaluating the freezing response time by inducing foot shock electrical stress. Training and testing sessions of fear conditioning were conducted in an inhibitory avoidance apparatus section (50 cm × 50 cm × 35 cm) with an inox steel rod floor (we did not use the dark side of shuttle box) attached to an electrical shock source.

The main outcome of interest was only freezing time response. Freezing was defined as the complete absence of discernable movements, except for respiratory movements. The reported freezing behavior was calculated by an experimenter blind to the groups as the proportion of total time freezing during the period each mouse was in the illuminated chamber on the last day of testing. On day 1, each mouse was placed in the illuminated compartment and was allowed to explore the testing environment for 5 min with the closed door between the sections. Afterward, the mice were returned to their home cage. On days 2–4, each animal was at first placed in the bright side of the apparatus while the door was open to enable the mouse to reach the dim partition within a period of 5 min. As soon as the mouse put in the box, a single electrical foot shock was delivered (50 Hz, 1.5 mA, for 2 s). On day 5, animals were put in the illuminated compartment for 5 min, and the freezing time for each mouse was measured, according to the aforementioned protocol.

### 2.6 Brain sample acquisition

In this section, after the behavioral experiment was completed, anesthesia is performed by CO_2_. After sacrificing animals, the brain was removed after cutting the parietal bone and optic nerve. Finally, the entire brain tissue lifted out and placed on ice, and carefully the hippocampal formation was removed and immediately placed in 4% polyformaldehyde solution for fixation or placed in liquid nitrogen for quick freezing, and then transferred to −80°C freezer for further use in gene expression using RT‐PCR. All samples that have been fixed in polyformaldehyde were sliced continuously with a microtome (thickness of 3 μm) for further evaluation.

### 2.7 Detection of oxidative stress indicators

The hippocampal tissue of each group was homogenized in 2 ml of phosphate buffered saline (PBS, pH 7.4), and then centrifuged at 12,000 × *g* for 20 min at 4°C for subsequent experiments. The determination of malondialdehyde (MDA), catalase (CAT), superoxide dismutase (SOD), and glutathione (GSH) were performed in accordance with the instructions of the kit (Beijing Solarbio Science & Technology Co., Ltd). In this experiment, the thiobarbituric acid (TBA) method was used to determine the content of MDA in hippocampus samples (Merás et al., 2020). Ammonium molybdate method was used to determine the activity of CAT, and the WST‐1 method was used to detect the activity of SOD content (Cao et al., 2021).

### 2.8 Immunofluorescence staining

Glial fibrillary acidic protein (GFAP) is an intermediate filament protein, which specifically expressed by astrocytes, and in this study, evaluation of GFAP was done to determine the level of astrocytes in hippocampus samples. For reaching this goal, after deparaffinizing the samples, they placed in an ethanol gradient solution (100%, 95%, then 85% and 75%, respectively); then floated in water for 5 min; then slices were placed in sodium borohydride solution, placed at room temperature for 30 min; and then rinsed with water for 5 min. We placed the samples form latest step in Sudan black dye solution, place them at room temperature for 5 min, and then rinse with water for 3 min and prepare samples for incubation with the primary antibody. We added the GFAP primary antibodies dropwise, which was diluted in appropriate proportions (the GFAP dilution ratio is 1:300), and incubated overnight at 4°C and then washed the samples with PBS solution 3 times on the next day. Then samples were incubated with the secondary antibody. In this regard, we added 50–100 μl of antimouse‐IgG‐labeled fluorescent antibody (GFAP is red fluorescence), and then incubated for 90 min in strict dark at room temperature, and then rinse 3 times with PBS solution. Also, we stained the nucleus with DAPI dye solution at room temperature for 10 min (under dark condition) and then rinsed with PBS, 3 times. Finally, all samples were observed under a fluorescence microscope and images were collected at 400× magnification.

### 2.9 RT‐PCR assessments

Total RNA was extracted from hippocampal tissue using TRIzol reagent (Invitrogen, Carlsbad, CA, USA) and was reverse‐transcribed into cDNA using a ReverTra Ace qPCR Kit (Toyobo, Osaka, Japan). Quantitative real‐time PCR (qPCR) was performed using an Ultra SYBR Mixture (CWBIO). The primers used in this study are as follows: forward primer of 5′‐CGGAGACGCATCACCTCTG‐3′ and reverse primer of 5′‐AGGGAGTGGAGGAGTCATTCG‐3′.

### 2.10 Statistical analysis

Statistical analysis was performed using GraphPad Prism 8 software (San Diego, CA, USA). The *p* value below .05 was regarded as significant. Since all data were normally distributed, one‐way ANOVA and two‐way ANOVA were used to compare the values between the groups. In addition, the sample size was determined using version 3 of G*Power software, considering the study's power of .8 and *α* = .05.

### 2.11 Ethics

Our study was in accordance with the National Institute of Health (NIH) Guidelines for the Care and Use of Laboratory Animals (HHS publication 85‐23, 1985), legislation for the protection of animals used for scientific purposes (Directive 2010/63/EU), and also this study was carried out in compliance with the ARRIVE guidelines (PLoS Bio 8(6), e1000412, 2010). All experimental protocols were approved by a Shahid Beheshti University of Medical Sciences and Xi'an Jiaotong University Health Science Center Ethics committee. In addition, in the current study euthanasia was done by using 7 min exposure to CO_2_.

## 3 RESULTS

### 3.1 Evaluation of learning and memory function in normal and socially isolated animals

In this section, we investigated the effect of SIS on learning and memory function by using MWM to elucidate if SIS could induce learning and memory impairment, in order to clarify the validity of SIS for further analysis. MWM experiment was used to detect and record the escape latency and swimming speed of the all groups of mice in the positioning navigation experiment (acquired training), and the percentage of residence time in the target quadrant (the quadrant where the original platform is located), and the escape latency and swimming speed of the mice in the visual platform experiment (as shown in Figures S1 and 1).

**FIGURE 1:**
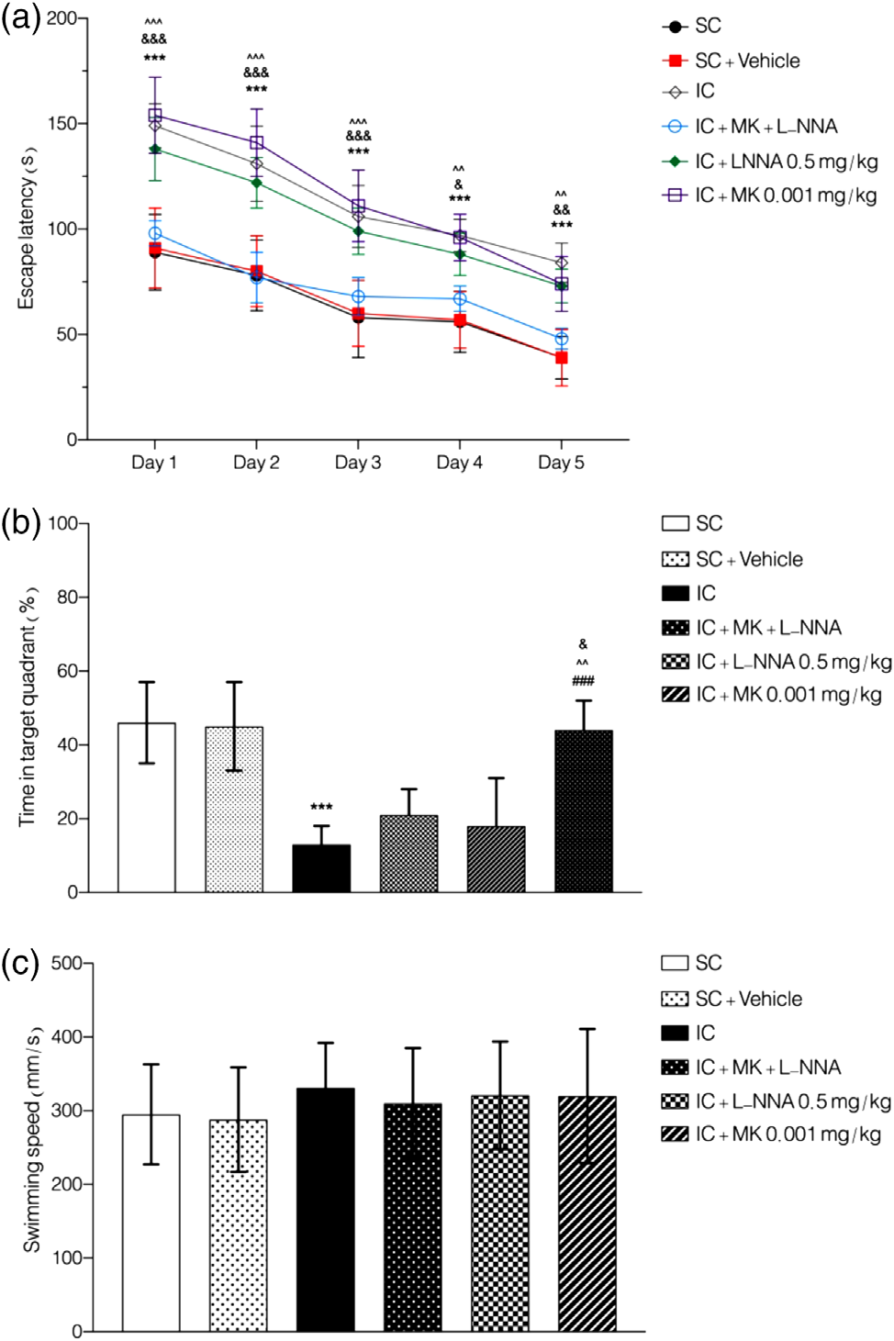
Effect of the SIS and combined treatment on learning and memory function. The combination therapy of MK‐801 and L‐NNA resulted in lower escape latency of Morris Water Maze (a) and higher time spent in the target quadrant. Values are expressed as the mean ± SEM (*n* = 5 in each group) results were analyzed using two‐way ANOVA repeated measure (a) and one‐way ANOVA (b and c) followed by Tukey's post hoc test. IC compared to SC: ***p < .001; combined treatment compared to IC animals ^###^ *p *< .001; combined treatment compared to IC+MK‐801: ^∧∧∧^ *p *< .001 and ^∧∧^ *p *< .01; combined treatment compared to IC+L‐NNA: ^&&&^ *p *< .01, ^&&^ *p *< .01, and ^&^ *p *< .05

two‐way ANOVA repeated measure analysis showed that none of the treatment groups used in this study could alter escape latency in normal animals (*F*(40, 220) = 0.328, *p >* .05, Figure S1b). In addition, two‐way ANOVA repeated measure analysis was done to find the effective and subeffective dose in each group of SIS. Our results showed that L‐NNA 1 mg/kg could significantly improve escape latency, but L‐NNA 0.5 mg/kg could not alter the escape latency (*F*(16, 115) = 0.481, *p <* .001, Figure S1c); thus, we chose L‐NNA 0.5 mg/kg as subeffective dose for further evaluation. In addition, MK‐801 (0.001 mg/kg, NMDAR antagonist) was used as subeffective dose (*F*(16, 115) = 0.952, *p <* .001, Figure S1d). Also, two‐way ANOVA repeated measure analysis failed to show any significant difference in socially isolated animals treated with NMDA (15 and 75 mg/kg, *F*(16, 100) = 0.518, *p >* .05, Figure S1e) and L‐arginine (50 and 100 mg/kg, *F*(16, 100) = 0.795, *p >* .05, Figure S1f) with nontreated isolation conditioned animals.

Also, we observed same results in evaluation of time spent in target quadrant. One‐way ANOVA analysis revealed no significant effect induced by any treatments on time spent in target quadrant in control group (*F*(10, 44) = 0.359, *p >* .05, Figure S2a). In addition, administration of NMDA at doses of 15 mg/kg and 75 mg/kg (*F*(4, 20) = 15.82, *p <* .001, Figure S2b) or L‐arginine at doses of 50 and 100 mg/kg (*F*(4, 20) = 17.40, *p <* .001, Figure S2e) in SIS group could not induce any significant effect on time spent in target quadrant in comparison to SIS group without any treatment. On the other hand, MK‐801 at 0.001 mg/kg (*F*(4, 20) = 8.86, *p <* .001, Figure S2c) and L‐NNA at 0.5 mg/kg (*F*(4, 20) = 12.31, *p <* .001, Figure S2d) were chosen as subeffective dose based on one‐way ANOVA analysis.

On the other hand, swimming speed was evaluated to declare that none of these drugs could induce any sedative or hyperactivity to distort the conclusion in this study. One‐way ANOVA analysis showed none significant effect on mice swimming speed after treating the animals with MK‐801, L‐NNA, NMDA, and L‐arginine in both normal and isolation contained animals (control groups (*F*(10, 44) = 0.215, *p >* .05, Figure S3a), MK‐801 administration (*F*(4, 10) = 0.305, *p >* .05, Figure S3c), NMDA administration (*F*(4, 10) = 0.310, *p >* .05, Figure S3b), L‐NNA administration (*F*(4, 10) = 0.475, *p >* .05, Figure S3d), and L‐arginine administration (*F*(4, 10) = 0.539, *p >* .05, Figure S3e).

The results of combination therapy with subeffective doses of MK‐801 (0.001 mg/kg) and L‐NNA (0.5 mg/kg) showed significantly shorter escape latency in comparison to socially isolated animals without any treatment (*F*(4, 135) = 106.8, *p <* .001, Figure 1a). Post hoc analysis showed that at days 1–5, combined treatment has significantly shorter latency‐time to reach the platform in comparison to SIS group (*p *< .001). Also, we observed that the group received a combined treatment of MK‐801 and L‐NNA had always a shorter escape latency time to reach the platform in days 1–5 in comparison to single dose administration of each MK‐801 or L‐NNA (*p *< .05).

One‐way ANOVA analysis revealed that combination treatment of MK‐801 and L‐NNA at their subeffective doses could significantly increase the time spent in target quadrant (*F*(5, 24) = 12.41, *p <* .001, Figure 1b). Tukey's test demonstrated that combination treatment of MK‐801 and L‐NNA significantly improved time spent in target quadrant in comparison to SIS group without any treatment and also MK‐801‐treated animals (in SIS group, *p *< .01) and L‐NNA‐treated animals (in SIS group *p *< .05). Besides, same as single treatment with MK‐801 or L‐NNA, combination treatment could not induce any significant effect on swimming speeds in socially isolated animals (*F*(5, 24) = 0.251, *p >* .05, Figure 1c). We also mentioned the representative swimming path tracings of mice in the training trial (days 1–5) in three major groups in this study.

In summary, the escape latency of SIS group was significantly prolonged, the percentage of time stayed in the target quadrant was significantly reduced, and the swimming speed was not changed in comparison to normal animals. These results are indicating that the learning and memory ability of socially isolated mice were significantly reduced, while treating animals with both L‐NNA and MK‐801 or their combination (at subeffective doses) can significantly shortening the escape latency and the percentage of time stayed in the target quadrant; furthermore, these data suggest that inhibition of NOS and NMDAR can improve the learning and memory impairment induced by SIS.

### 3.2 Evaluation of fear memory extinction in normal and socially isolated animals

In this section, we assessed the freezing response in order to evaluate the fear memory extinction in both SIS and normal group. In the first step, we tried to elucidate the effective and subeffect doses for L‐NNA and MK‐801 (Figure S4). Interestingly, same as previous part, our results showed that MK‐801 at 0.001 mg/kg (*F*(4, 20) = 41.55, *p <* .001, Figure S4c) and L‐NNA at 0.5 mg/kg (*F*(4, 20) = 55.71, *p <* .001, Figure S4d) exerted subeffective dose; also, administration of NMDA (*F*(4, 20) = 39.84, *p <* .001, Figure S4b) and L‐arginine (*F*(4, 20) = 42.54, *p <* .001, Figure S4e) could not induce any significant effect on freezing response. Also, we observed that none of the treatments had a significant effect on freezing time in normal conditioned animals (*F*(10, 44) = 0.094, *p >* .05, Figure S4a).

**FIGURE 2:**
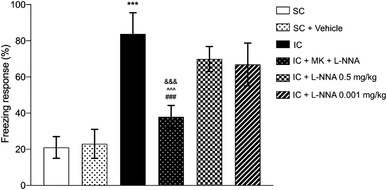
Effect of the SIS and combined treatment on function of fear memory extinction. The combination therapy of MK‐801 and L‐NNA resulted in lower freezing response. Values are expressed as the mean ± SEM (*n* = 5 in each group). Results were analyzed using one‐way ANOVA followed by Tukey's post hoc test. IC compared to SC: ***p < .001; combined treatment compared to IC animals ^###^ *p *< .001; combined treatment compared to IC+MK‐801: ^∧∧∧^ *p *< .001; combined treatment compared to IC+L‐NNA: ^&&&^ *p *< .01

On the other hand, one‐way ANOVA analysis demonstrated that administration of combined MK‐801 and L‐NNA at subeffective doses in socially isolated animals could significantly decrease the freezing response in comparison to SIS group (*F*(5, 24) = 45.82, *p <* .001, Figure 2). Tukey's analysis exhibited that administration of MK‐801 at dose of 0.001 mg/kg/day in combination with L‐NNA at dose of 0.5 mg/kg significantly reduced the freezing behavior in IC mice compared to IC vehicle group (*p <* .001). Moreover, IC mice that received the combination treatment had significantly lower freezing response in comparison to single‐treatment of L‐NNA and MK‐801 (*p *< .001). Interestingly, no significant difference was observed between combined therapy and social conditioned (*p >* .05).

### 3.3 Blockage of NO/NMDAR pathway can modulate the oxidative stress and expression of GFAP in socially isolated animals

**FIGURE 3:**
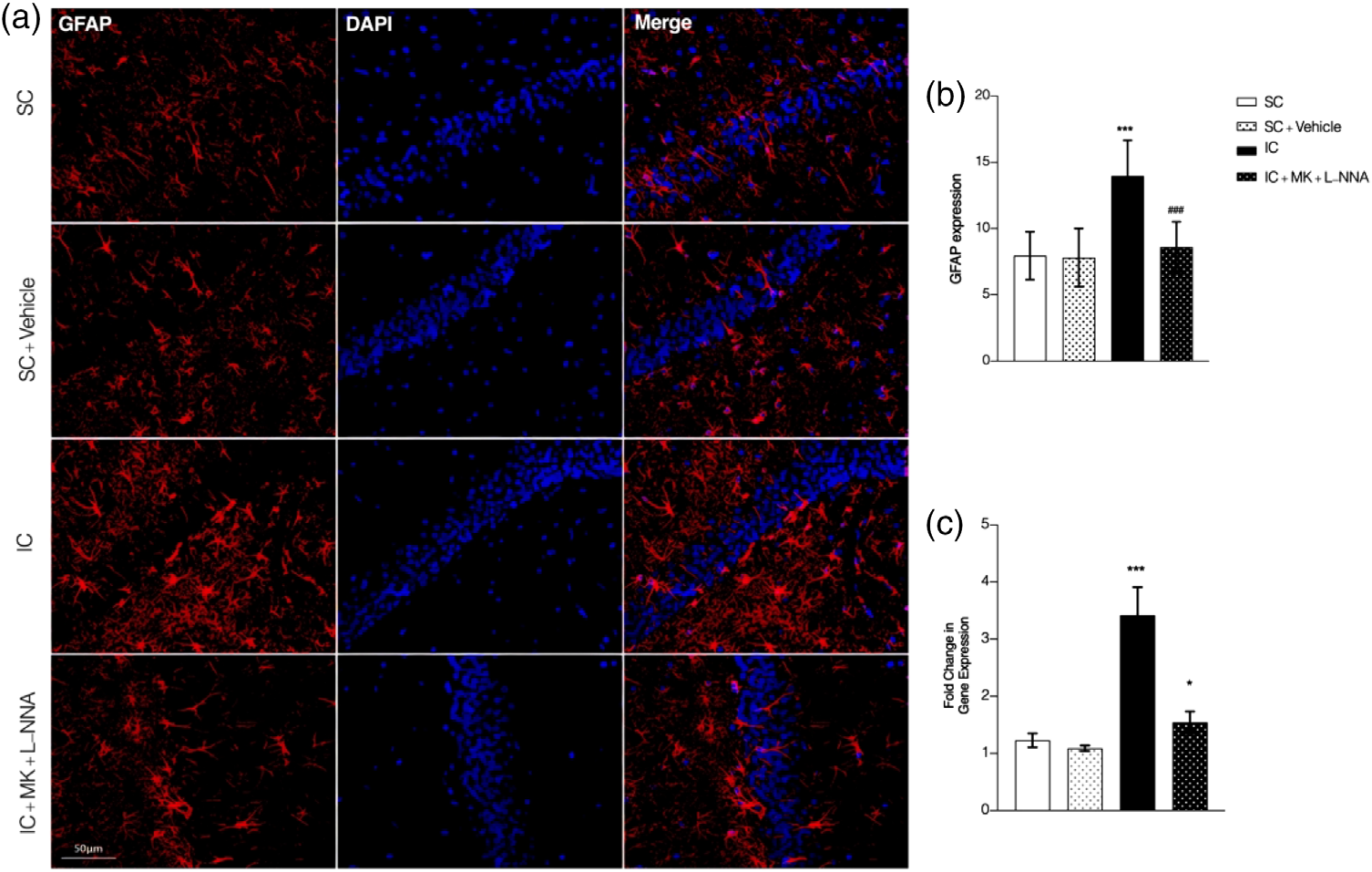
Immunohistochemistry (a and b) and gene expression evaluation using RT‐PCR (c) of GFAP. The combination therapy of MK‐801 and L‐NNA resulted in lower GFAP expression. Values are expressed as the mean ± SEM (*n* = 5 in each group). Results were analyzed using one‐way ANOVA followed by Tukey's post hoc test. IC compared to SC: ***p < .001 and *p < .05; combined treatment compared to IC animals ^###^ *p *< .001 and ^#^ *p *< .05

In order to clarify the effect of combination therapy of MK‐801 and L‐NNA on the activation of astrocytes in socially isolated mice, immunofluorescence staining and RT‐PCR methods were used to detect the expression of astrocyte marker GFAP in hippocampus (as shown in Figure 3a and b).

The results of immunofluorescence staining showed that the GFAP fluorescence expression in the hippocampus of the socially isolated animals was increased in comparison to the control group (*F*(3, 16) = 9.109, *p <* .001, Figure 3b); however, the GFAP fluorescence expression of the socially isolated animals treated with combination of MK‐801 (0.001 mg/kg) and L‐NNA (0.5 mg/kg) was significantly decreased in comparison to SIS group (*p <* .001). In addition, the comparison of combined therapy with the control group, showed no statistically significant changes (*p >* .05). These results suggested that the increased hippocampal astrocytes induced by SIS could be regulated by MK‐801 in combination with L‐NNA.

On the other hand, gene expression results demonstrated that the GFAP protein expression in the socially isolated animals was significantly increased (*F*(3, 16) = 28.61, *p <* .001, Figure 3c) in comparison to normal animals. Post hoc analysis showed that the GFAP protein expression in the isolation conditioned mice treated with combined treatment was significantly reduced in comparison to socially isolated animals without any treatment (*p *< .05). Also, in comparison with the control group, isolation conditioned mice treated with combined treatment showed a slightly significant difference (*p *< .05). The above results indicate that astrocytes in socially isolated mice, which were significantly activated, and MK‐801 plus L‐NNA could reduce the activation of astrocytes in these animals.

### 3.4 Blockage of NO/NMDAR pathway can modulate the oxidative stress in socially isolated animals

**FIGURE 4:**
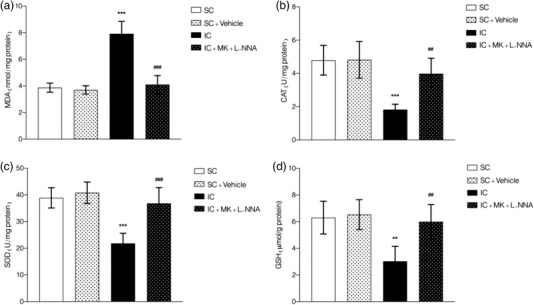
Evaluation of oxidative stress components in hippocampal tissue in both normal and socially isolated animals. The combination therapy of MK‐801 and L‐NNA resulted in lower MDA (a), higher CAT (b), higher SOD (c), and higher GSH (d) in comparison to socially isolated animals. Values are expressed as the mean ± SEM (*n* = 5 in each group). Results were analyzed using one‐way ANOVA followed by Tukey's post hoc test. IC compared to SC: ***p < .001 and **p < .01; combined treatment compared to IC animals ^###^ *p *< .001 and ^##^ *p *< .01

In order to clarify the effect of combined treatment of MK‐801 and L‐NNA on oxidative stress in socially isolated animals, the hippocampal tissues of each group were determined by the thiobarbital method to determine the content of malondialdehyde (MDA); also, the ammonium molybdate method was used to determine catalase (CAT) activity. In addition, WST‐1 method was used to detect the activity of superoxide dismutase (SOD), and microenzyme method was used to determine the content of glutathione (GSH) (as shown in Figure 4).

The results showed that the content of MDA in the hippocampus of socially isolated animals was significantly increased (*F*(3, 16) = 53.35, *p <* .001, Figure 4a) in comparison to normal mice, and the activity of CAT (*F*(3, 16) = 13.33, *p <* .001, Figure 4b), SOD (*F*(3, 16) = 9.436, *p <* .001, Figure 4c), and the content of GSH (*F*(3, 16) = 18.79, *p <* .001, Figure 4d) were significantly reduced in comparison to normal mice. Post hoc analysis showed that the content of MDA in hippocampal tissue obtained from socially isolated animals treated with MK‐801 and L‐NNA was significantly reduced in comparison to isolation contained animals (*p <* .001) and also the activity of CAT, SOD, and GSH were significantly increased (*p <* .01, *p <* .001, and *p <* .01, respectively). In addition, in comparison with the control group, the content of MDA and the activity of CAT, SOD, and GSH in the hippocampus were not significantly changed after coadministrating of MK‐801 and L‐NNA (*p *> .05). There results showed that the oxidative stress induced by SIS could be regulated by combined treatment of MK‐801 and L‐NNA.

### 3.5 Correlation analysis of freezing response, GFAP expression, and oxidative stress markers

**FIGURE 5:**
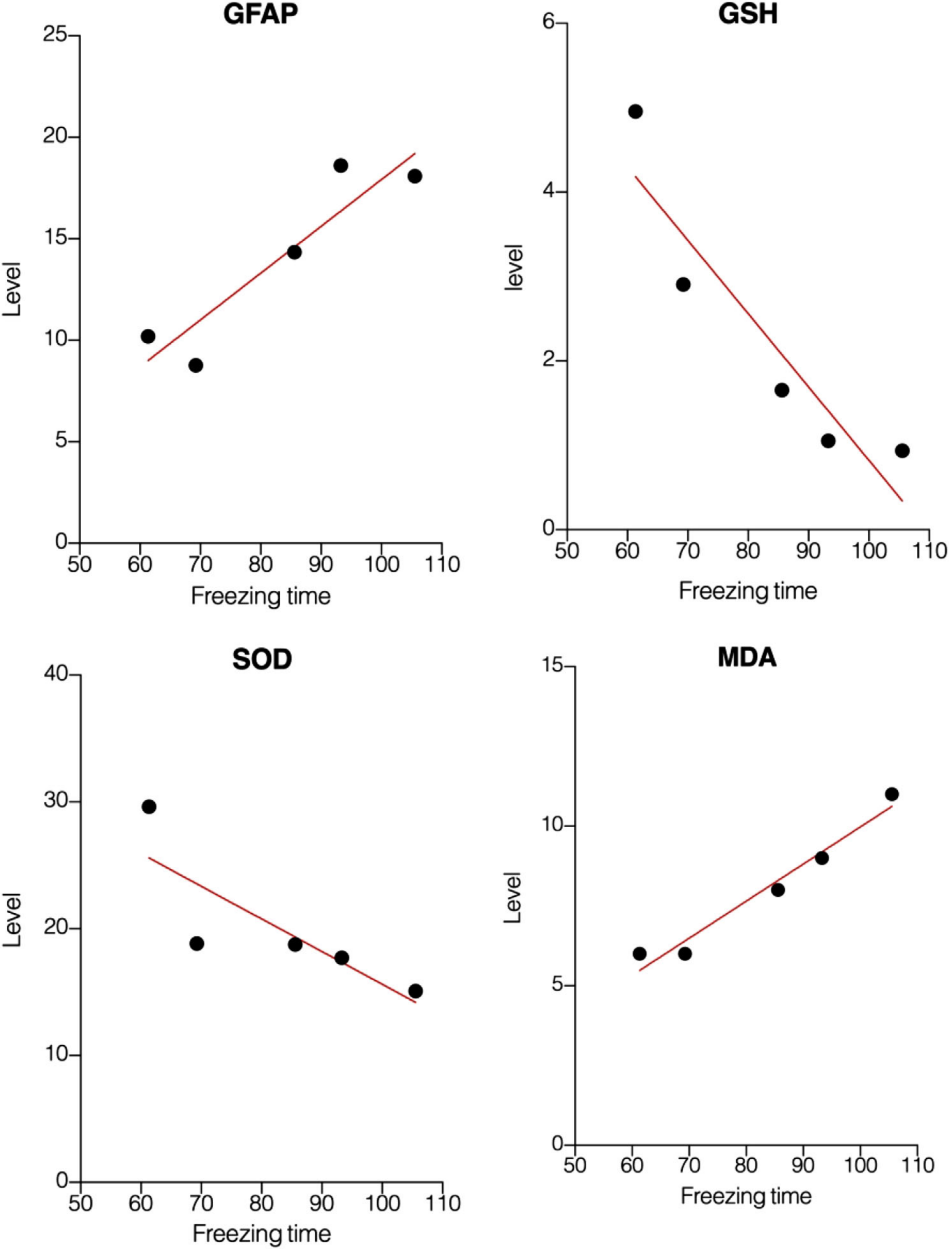
Correlation analysis of freezing response, GFAP expression, and oxidative stress markers

In this section, we used correlation analysis to evaluate the possible relation between oxidative stress, astrocyte activity, and freezing response in socially isolated animals (Figure 5). Our results demonstrated that higher GFAP expression and higher level of MDA were significantly correlated with higher freezing time (*p = *.0258, *r* = .922 and *p = *.003, *r* = .980, respectively). In addition, GSH level was indirectly correlated with freezing response time (*p = *.023, *r* = −.926). However, we did not observe any significant correlation between the level of SOD and freezing response time in socially isolated animals (*p = *.08, *r* = −.825).

## 4 DISCUSSION

In the current study, we observed that SIS could significantly induce learning and memory dysfunction as well as impairment of fear memory extinction in MWM and freezing response tests. Also, we observed that combined treatment including blockage of NOS (by L‐NNA, 0.5 mg/kg) and NMDAR (by MK‐801, 0.001 mg/kg) at subeffective doses could result in improvement of both learning and memory function and fear memory extinction. In addition, we observed that SIS significantly increases the GFAP expression and astrocyte activity, which results in significant imbalance in oxidative stress. Coadministration of MK‐801 and L‐NNA at subeffective doses not only decreases the expression of GFAP but also regulates the oxidative stress imbalance.

Previously, it has been well‐documented that SIS could result in learning and memory impairment (Holt‐Lunstad et al., 2010), (McLean et al., 2010) (Yusufishaq & Rosenkranz, 2013; Weiss et al., 2004). In this regard, Chida et al. (2006) showed that chronic SIS exacerbated conditioning memory in mice, which could be probably through a glucocorticoid‐mediated decrease in neural activation. In addition, Frisone et al. (2002) demonstrated that SIS could significantly impair the spatial memory in rodents. Although, in the current study, we investigated the spatial memory by using MWM for validating the SIS mode, our results are in line with previous studies. We observed that SIS could significantly impair spatial memory in mice by using MWM.

On the other hand, numerous studies elucidated that SIS could significantly increase freezing time (PTSD‐like behavior) in rodents, and SIS was introduced as a valid model for PTSD in animal. In this regard, Lukkes et al. (2009) and Zelikowsky et al. (2018) investigated effects of SIS on PTSD‐like behavior and freezing response. They have showed that SIS could induce fear memory extinction impairment after social isolation stress (). In line with previous studies, we showed that baseline freezing response in SIS group was significantly increased.

It has been shown that NMDAR is necessary for excitatory and inhibitory synaptic transmission (Cull‐Candy et al., 2001). Yang et al. (2018) found that the expression of NMDAR‐2B subunit hippocampal tissue was significantly decreased in animals exposed to chronic stress. In contrary, Calabrese et al. (2012) have shown that prolonged stress enhances NR1 and NR2B subunit expression in rat hippocampus. Although, various factors such as animal model might contribute to the differences, they showed that NMDAR is significantly involved in fear memory extinction in animals exposed to stress. In addition, it has been demonstrated that ketamine (NMDAR antagonist) might attenuate the decrease of NMDA receptor‐mediated excitatory postsynaptic currents and the density of NR2B in the hippocampus, which might underlie the ability why ketamine can alleviate the memory dysfunction induced by chronic stress (Yang et al., 2018). Our results are in line with the previous studies; in the current study, we observed that blockage of NMDAR could significantly improve the learning and memory function and also improve fear memory extinction in socially isolated animals.

The interaction between NO (nitric oxide) and NMDA in learning and memory process has been widely reviewed. In the current study, we observed that learning and memory impairment as well as dysfunction in fear memory extinction induced by SIS were significantly improve after coadministration of MK‐801 (NMDAR antagonist) and L‐NNA (NOS inhibitor). These results suggest a synergic effect through probably a same underlying mechanism. Besides, Reyes et al. (2012) showed that activation of neural NMDAR might lead to an imbalance in oxidative stress and consequently cause neighboring neurons death. In addition, interestingly they showed that this process might be caused by upregulation and high activity of astrocytes. Thus, we hypothesized that NO/NMDAR pathway might be a novel underlying mechanism in formation of PTSD‐like behavior following social isolation stress.

Astrocytes are one of the most widely distributed cells in the brain, which play an important role in the structure and function of the brain (Dong & Benveniste, 2001). Astrocytes have the functions of supporting and separating neurons, participating in the formation of the blood–brain barrier, transmitter metabolism, transmitting biological information, and maintaining ion balance around neurons and may participate in a variety of neuropathological processes including neuroinflammation (Dong & Benveniste, 2001; Liddelow & Barres, 2017). Previous evidence has shown that astrocytes might be upregulated and activated in rodents exposed to stress (Mayhew et al., 2015). Also, it has been well‐documented that activation and proliferation are usually accompanied by increased expression of glial fibrillary acidic protein (GFAP, a marker of astrocytes) (Brenner et al., 1994). Therefore, the increase of GFAP in the tissue is a manifestation of astrocyte activation, and it is also a landmark signal of the central nervous system's response to pathophysiological condition (Brenner et al., 1994). In addition, previous studies showed that SIS could increase the oxidative stress in hippocampus and might resulted in neural death. In this regard, the underlying mechanism suggested that when a cell is irreparably injured, a signaling cascade is triggered, which might result in cell death. Numerous routes contribute to the activation of apoptosis in neural cells, but one of the most important is the generation and spread of free ROS. The generation of ROS has been associated with the activation of nicotinamide adenine dinucleotide phosphate (NADPH) oxidase (Lucke‐Wold et al., 2015). NADPH oxidase (NOX) contributes to cellular host defense in the absence of damage via cytokine signaling, gene expression control, posttranslational protein processing, endoplasmic reticulum stress response, and tissue homeostasis. Astrocytes highly express NOX isoforms and the NOX pathway contributes to ROS generation only in response to damage or illness (Lucke‐Wold et al., 2015). In this study, we hypothesize that higher activation of astrocytes might resulted in high ROS formation. Our results demonstrated that the level of SOD and GSH were significantly increased in animals faced SIS, which can provide an evidence that activation of astrocytes following SIS can result in high ROS formation and neural damage.

In the current study, the results of immunofluorescence staining and gene expression in this study showed that compared with the control group, the expression of GFAP in the SIS group increased and compared with the SIS group, the expression of GFAP in the combined treatment group (MK‐801 plus L‐NNA) decreased. These results are indicating that astrocytes might be activated following SIS and coadministration of MK‐801 and L‐NNA can inhibit the activation of astrocytes and also inhibit the subsequent consequences. Besides, we observed that combined treatment could regulate the imbalance oxidative stress induced by SIS. Further studies need to be established to evaluate the role neuroinflammation and the underlying mechanism of microglia and astrocyte in PTSD‐like behavior inducing by socially isolation stress. In the current study, evaluation of HPA‐axis dysfunction by evaluation the plasma corticosterone level, hyperarousal, and fear extinction impairments might help to better understanding the underlying mechanism of PTSD‐like behavior in socially isolation animals, and unfortunately, in this study, due to limitation we were not able to evaluate these items. Finally, this study suffers from several limitations as follows. Although the results of this study suggest that blocking NO/NMDA pathway resulted in modulation of astrocyte activity, there is no direct evidence that this process resulted in reduced oxidative stress or high oxidative stress level following other underlying mechanisms impact on NO/NMDA pathway activation. Thus, we suggest future study to evaluate this phenomenon in NMDA knockout animals to clarify this question. In addition, the role microglia in PTSD‐like behavior induced by SIS should be investigated in future researches. Besides, due to some financial issues, this study could not provide any data of NOS isoforms expression.

## 5 CONCLUSION

In conclusion, impaired learning and memory function as well as fear memory extinction induced by SIS might be exerted through NO/NMDAR pathway and activation of astrocytes and activation of astrocytes might result in oxidative stress imbalance. Based on this study, it could be hypothesized that blockage of NO/NMDAR pathway might be a novel treatment for PTSD‐like behavior in animals by inhibiting the astrocyte and regulating oxidative stress level.

## CONFLICT OF INTEREST

There is no conflict of interest to declare.

### PEER REVIEW

The peer review history for this article is available at https://publons.com/publon/10.1002/brb3.2620

## Supporting information

Supporting information

## Data Availability

The datasets generated and/or analyzed during the current study will be available on request to corresponding authors.
