# Supplementary material for: Modulation of astrocyte activity and improvement of oxidative stress through blockage of NO/NMDAR pathway improve posttraumatic stress disorder (PTSD)‐like behavior induced by social isolation stress
Source: Brain Behav. 2022 May 23;12(7):e2620. doi: 10.1002/brb3.2620 (PMC9304825; doi:10.1002/brb3.2620)
Supplement: Supplementary file 1 — Supporting information [file BRB3-12-e2620-s001.docx]

**Supplementary figures and captions**

**
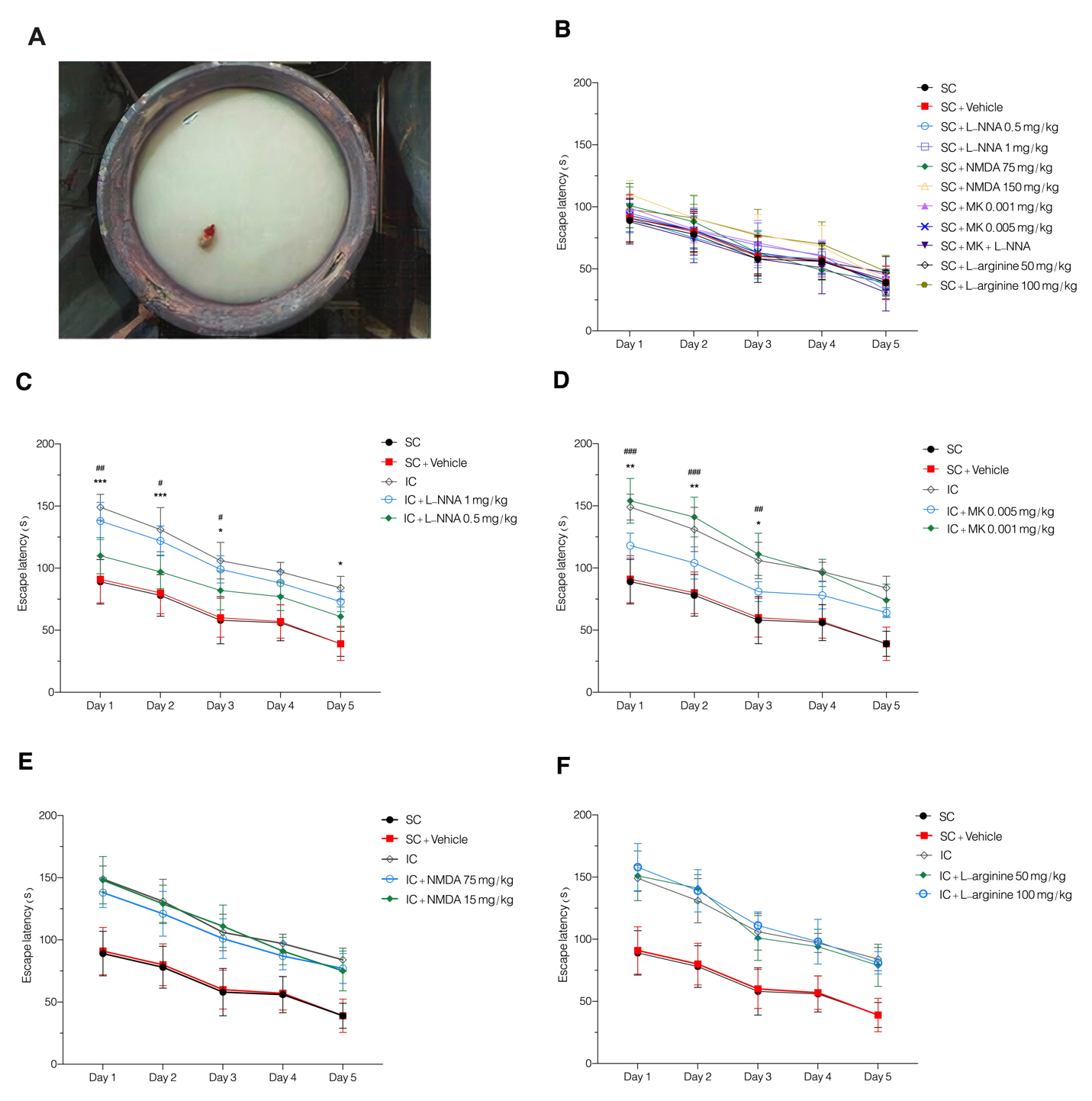
**

**Figure 1S. The comparison of the time of the escape latency (Morris Water Maze) in all treatment groups.**

**
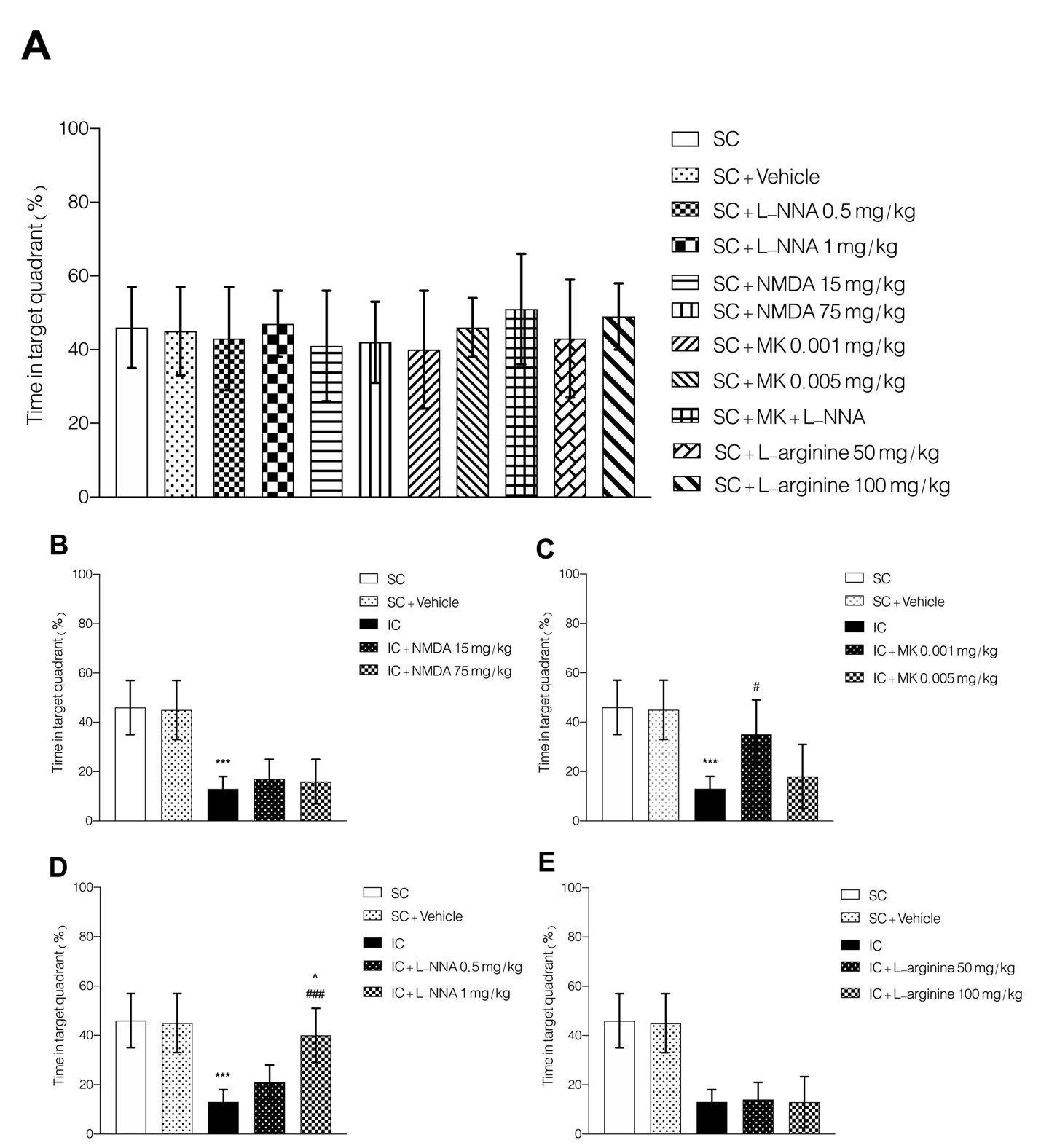
**

**Figure 2S. The comparison of the ratio of time spent in the target quadrant to total time (Morris Water Maze) in all treatment groups.**

**
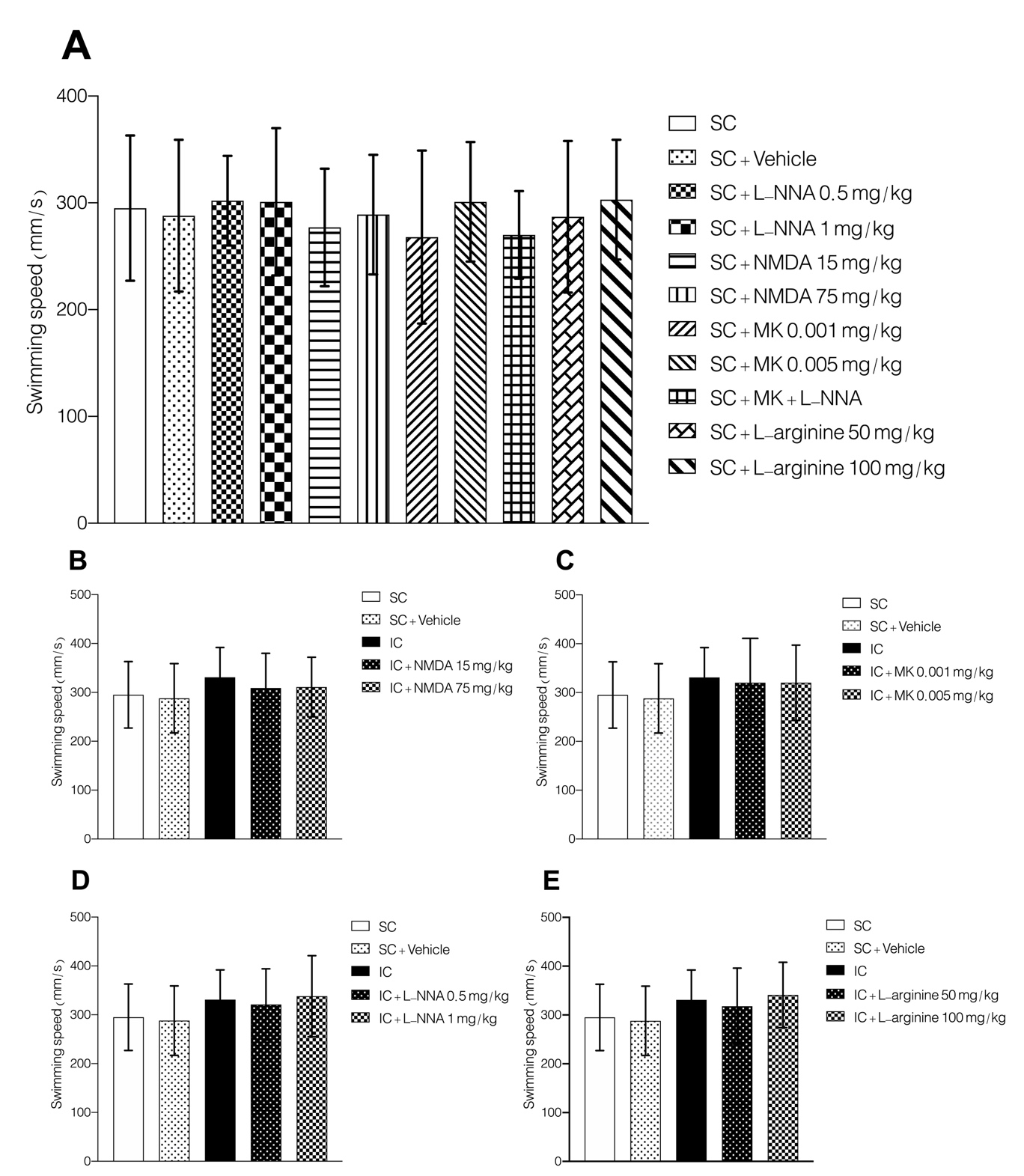
**

**Figure 3S. The comparison of the swimming speed (Morris Water Maze) in all treatment groups.**

**
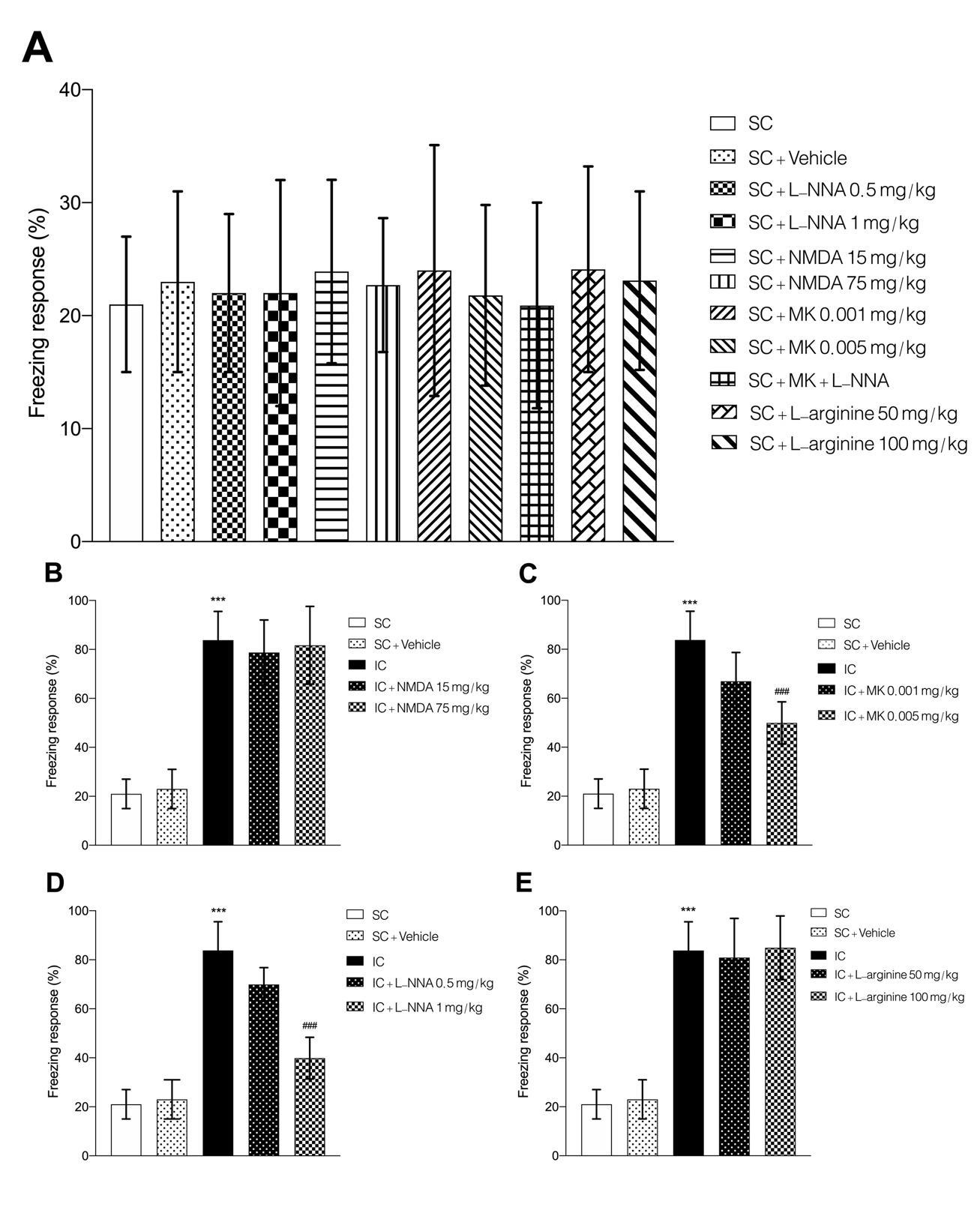
**

**Figure 4S. The comparison of the freezing time in all treatment groups.**
